# Deformation monitoring and safety stability evaluation study of high-altitude limestone dumps

**DOI:** 10.1371/journal.pone.0318589

**Published:** 2025-02-18

**Authors:** Jianjun Dong, Yawen Guo, Yuan Mei, Ke Gao

**Affiliations:** 1 College of Safety Science and Engineering, Liaoning Technical University, Huludao, Liaoning, China; 2 Key Laboratory of Mine Thermodynamic Disasters and Control of Ministry of Education, Liaoning Technical University, Huludao, Liaoning, China; Makerere University College of Natural Sciences, UGANDA

## Abstract

We employed synthetic aperture radar interferometry (InSAR) to assess the slope stability of a high-altitude landfill in Sangri County, Shannan, Tibet. To address the unique climatic conditions of high-altitude regions, the InSAR deformation monitoring model was enhanced to mitigate the effects of temperature and rainfall. The accuracy of InSAR monitoring in high-elevation slopes was validated by comparison with GNSS RTK measurements. Both Differential Interferometric Synthetic Aperture Radar (D-InSAR) and Small Baseline Subset Interferometric Synthetic Aperture Radar (SBAS-InSAR) techniques were applied to monitor and evaluate slope deformation at the landfill site. The findings indicate that the average error between the improved InSAR model and GNSS measurements is 0.28 mm, with no statistically significant difference. The maximum slope displacement exceeds 20 mm when rainfall exceeds 300 mm, reaching the blue warning threshold. From 2018 to 2022, the deformation rate of the high-altitude landfill ranged from 0 to − 9.00 mm/a, classified as slip category VII. Significant deformation was observed during the rainy season, while the slope remained stable during dry periods, suggesting that rainfall is a primary trigger for slope deformation. A certain hysteresis effect in the deformation response to rainfall was also identified. The results demonstrate that InSAR technology offers comprehensive and dynamic monitoring capabilities for high-altitude slopes and serves as an effective tool for slope stability management in challenging environments.

## Introduction

The dump monitored in this study is in Sangri County, Shannan, Tibet, a high-altitude region characterized by intense plateau erosion. This harsh environment complicates the preservation of continuous Quaternary loose sediments, making the area highly susceptible to landslides that pose significant risks to mining operations [1]. Effective monitoring of slope deformation in this region is, therefore, crucial for disaster prevention and operational safety.

Traditional subsidence monitoring techniques, such as precise leveling, are labor-intensive, time-consuming, and expensive [2]. Open-pit mining exacerbates slope deformation and instability, further complicating monitoring efforts. Although the Global Positioning System (GPS) has been employed in slope monitoring, it is limited in handling large-scale tasks and often fails to provide high accuracy for vertical displacement measurements [3].

Interferometric Synthetic Aperture Radar (InSAR) has emerged as a high-precision remote sensing technology for deformation monitoring, achieving millimeter-level accuracy. InSAR has shown considerable potential for detecting ground movements, including surface subsidence, landslides, seismic deformation, volcanic activity, and glacial drift, as well as monitoring subtle displacements in structures such as bridges, dams, and buildings [4–8]. By combining near real-time monitoring, cloud penetration, and three-dimensional spatial consistency, InSAR offers significant advantages over traditional techniques. Its ability to monitor large areas remotely makes it ideal for long-term observation of high-altitude dump slopes. InSAR also addresses the logistical challenges of instrument deployment and fieldwork in such regions, where high costs and risks—such as altitude sickness and severe hypoxia—often impede conventional monitoring methods. This makes comprehensive and stable monitoring of high-altitude dump slopes both feasible and reliable.

The Small Baseline Subset InSAR (SBAS-InSAR) technique has proven particularly effective for monitoring slope deformation and identifying potential landslide risks [9]. Necsoiu et al. employed multi-temporal SBAS-InSAR to analyze landslide displacement rates in Salmon Falls Creek Canyon, Idaho, demonstrating its ability to capture nonlinear displacement patterns and validate results with GPS measurements [10]. Qu et al. used the SBAS-InSAR technique to monitor a large-scale landslide in Li County, Sichuan, revealing deformation rates of up to 150 mm/year, validated by in-situ inclinnometer data, thus providing reliable support for landslide monitoring and early warning [11]. Tian et al. applied SBAS-InSAR to monitor landslides in eastern Qinghai Province, identifying 491 hazardous landslides with deformation rates up to 298 mm/year, offering new insights into landslide mechanisms and their relationship with precipitation [12]. Guo et al. used SBAS-InSAR to analyze the deformation of the Xiongba ancient landslide, revealing two large deformation zones with cumulative displacements of 204 mm and 302 mm, respectively, and highlighting the influence of nearby landslide hazard chains on deformation acceleration [13]. Rehman et al. employed SBAS-InSAR to investigate landslide activity in the Hunza-Nagar region, identifying four active landslides with deformation rates ranging from − 300 mm/year to 20 mm/year [14]. Hu et al. applied SBAS-InSAR to detect ground deformation along the Longqing Highway, identifying six potential slope hazards that were verified through field surveys, demonstrating the technique's effectiveness for regional highway slope inspection [15].

Global Navigation Satellite System (GNSS) technology, a robust all-weather satellite navigation and positioning system, is also widely used for slope monitoring. GNSS offers variable positioning accuracies ranging from millimeters to meters. Notable studies include Hu et al., who combined GNSS with automated inclinometer systems to identify active deformation zones in Qinghai, China, and employed SPH modeling to predict dynamic hazards [16]. Shen et al. developed a GNSS real-time kinematic positioning method for detecting abrupt landslide displacements, validated through simulations and field experiments, providing a novel framework for short-term monitoring [17]. Cenni et al. integrated archival aerial photogrammetry, GNSS, and InSAR data to monitor the Patigno landslide in Italy over 44 years, revealing correlations between rainfall and sliding accelerations [18]. Despite the high accuracy of GNSS, the difficulty of manual point deployment and the impact of high-density point deployment on slope stability remain significant challenges.

While previous studies have applied SBAS-InSAR to monitor slope deformation, they have generally overlooked the effects of meteorological factors such as rainfall and temperature on the InSAR deformation monitoring model. Furthermore, they have not analyzed the relationship between rainfall and landslide deformation. This paper, therefore, improves the InSAR deformation monitoring model by eliminating the influence of meteorological factors on the inter-seasonal calculation of SBAS-InSAR data. It also elucidates the hysteresis effect of rainfall on slope deformation, based on rainfall and deformation monitoring data.

In this study, we focus on high-elevation dumping slopes in Sangri County, Shannan Region, Tibet, and monitor deformation from November 2018 to October 2022 using InSAR technology. The reliability of the SBAS-InSAR monitoring results was improved by refining the InSAR deformation model to account for climatic factors such as rainfall and temperature. A safety and stability evaluation of the slope at the discharge site was conducted based on the deformation monitoring results. Additionally, we analyze the relationship between rainfall and deformation patterns, providing theoretical and technical support for the safety and stability of dump slopes in high-altitude mining areas.

## Study area and data description

### General situation of high-altitude limestone dumps

**Fig 1:**
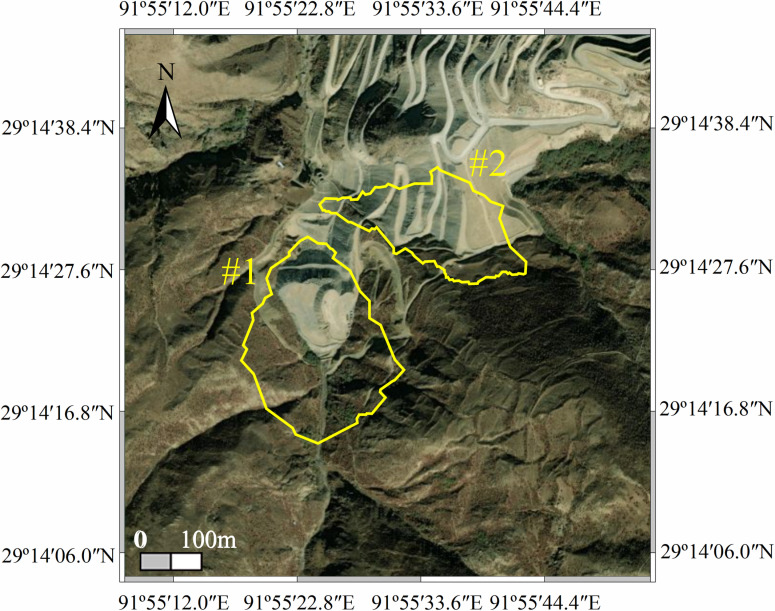
Slope location map of the dump.

The dump slopes are crucial auxiliary structures in mining operations. The dump area is divided into two sections: Dump No. 1 and Dump No. 2, located in two gullies on the southern side of the open-pit mining site. These two dumps are separated by a ridge, as shown in Fig 1.

The study area is situated in an open-pit mining zone in Sangri County, Tibet Autonomous Region, behind the Huaxin (Tibet) Cement Plant in Mamayi Township. It lies in the southern part of the middle Yarlung Zangbo River Valley, on the southern edge of the eastern section of the Gangdise Mountains. The terrain is characterized by a plateau and high mountain-wide valley type, with a general slope from south to north. Elevations in the region range from 3,539 m to 4,860 m. Mining operations are ongoing, and no geological disasters have been reported to date. The surrounding area hosts residential populations, the Lalin Railway, and the Yarlung Zangbo River, necessitating high safety standards for the slopes within the mining area. The residential area is located downstream. Dump No. 1 has an area of 111,340 m^2^, while Dump No. 2 covers 68,660 m^2^.

**Fig 2:**
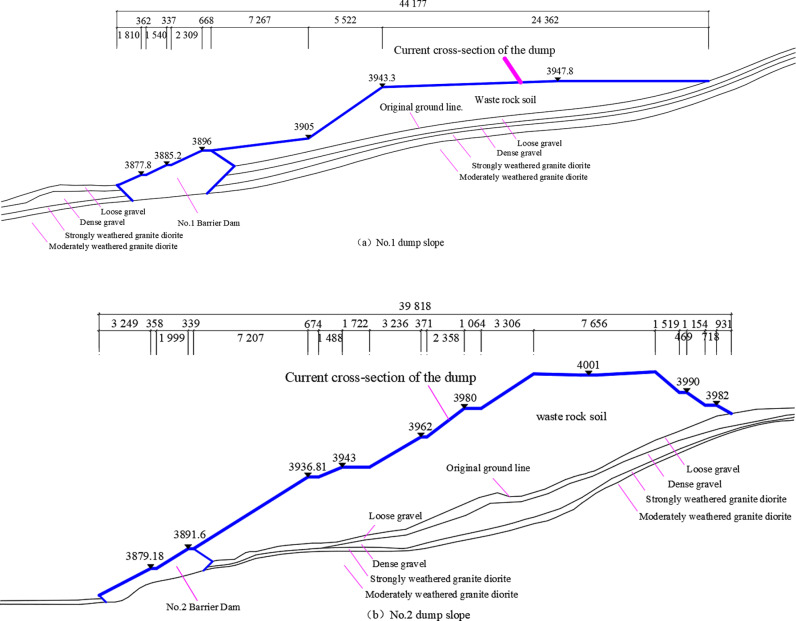
Profile of dump slope in-service.

According to the survey data, the soil dump area exhibits a vertical stratification, consisting of three primary layers (Fig 2). The first layer, Q4ml, or artificial fill layer, primarily consists of powdery clay and discarded limestone blocks. This layer forms the principal component of the dump slope. The second layer, Q4al+pl, or Quaternary alluvial layer, is made up of crushed stone soil, which is divided into two sub-layers of loosely and slightly densely packed crushed stones, depending on the degree of compaction. The content of slightly dense crushed stones ranges from 55–65%, and their particle sizes generally range from 2–15 cm, with a small proportion larger than 15 cm. This layer consists of granite, feldspar, and sandstone, which are distributed throughout the mountainous region. The third layer, γδK1, or Early Cretaceous, represents the entire dumping area and is primarily composed of granite rock. This layer is classified into strongly weathered and moderately weathered granite, based on the degree of weathering.

### Distribution characteristics of rainfall during rainy season in high-altitude area

The climate in Sangri County is cold and dry during winter, with significant diurnal temperature fluctuations. Summers are warm and humid, and there are distinct wet and dry seasons. The area experiences frequent and abrupt weather changes, with an average annual temperature of 1.3°C. January is the coldest month, with an average temperature of −9.9°C, while July is the warmest, with an average temperature of 10.8°C. The temperature variation throughout the year is 21.7°C, and the maximum daily temperature difference is 18.0°C. The region receives an average annual precipitation of approximately 500 mm, with 85% of it occurring between June and August. The average annual evaporation rate is 1,725.7 mm. Natural disasters such as snowstorms, hail, frost, droughts, and strong winds are common. The region is classified as having a high-altitude, cold-temperate, semi-arid monsoon climate. Winters are cold, summers are cool, and strong winds are frequent, with winds of level 8 or higher occurring for an average of 74 days per year. The most frequent natural disaster is snow, followed by wind and drought.

Due to the unique geological conditions and the harsh natural environment of the high-altitude area, the dump slopes and surrounding mountains are in a loose state. The combination of high altitude and severe weathering results in poor integrity of both the dumps and the surrounding mountains. The shear strength of the dump slope and the surrounding soil decreases under the influence of rainfall, making the area prone to landslides and other disasters.

**Fig 3:**
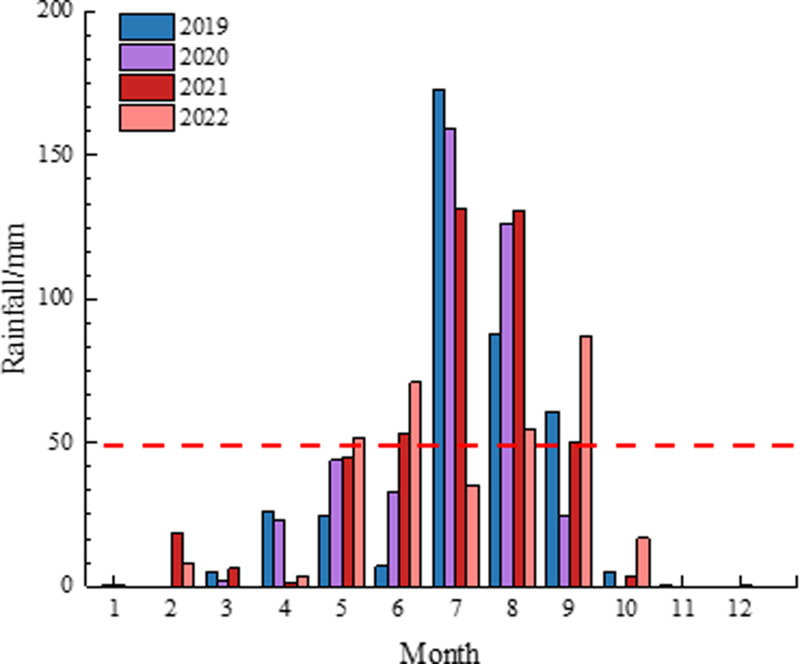
Monthly rainfall from 2019 to 2021.

**Fig 4:**
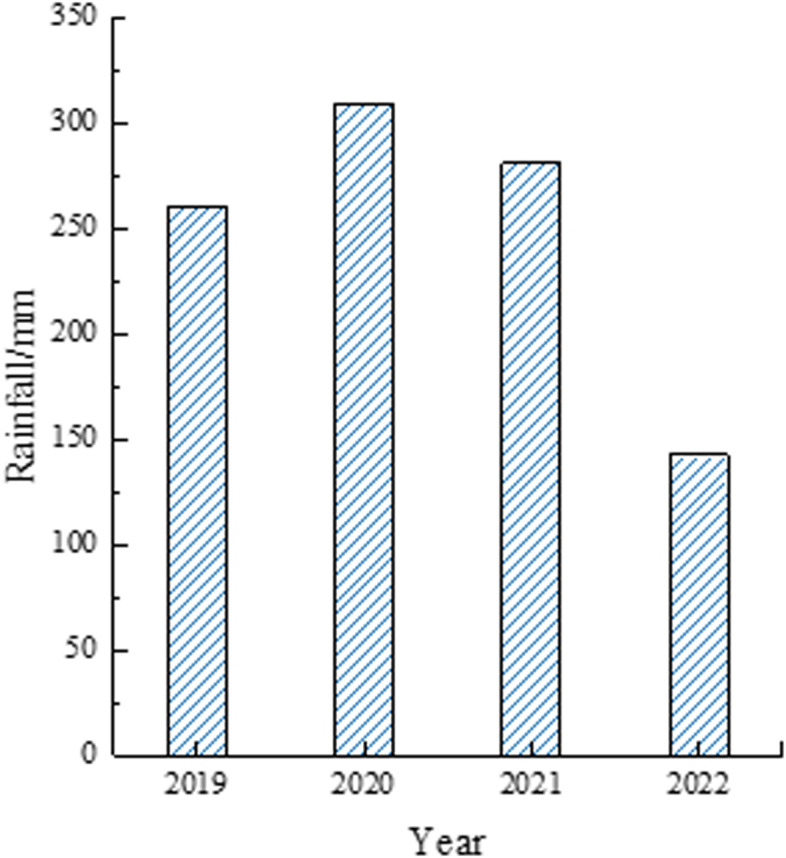
Rainfall during flood season.

During rainfall, shallow landslides induced by heavy or torrential rain are common and frequent geological phenomena, often considered disasters. Therefore, deformation monitoring image data was selected by analyzing the monthly rainfall from 2019 to 2022 (Fig 3). Sentinel image data, collected in two scenes per year, were used, with data from the rainy season (July to September) selected for analysis. The total rainfall during this period was calculated and is shown in Fig 4.

### Deformation monitoring data selection

Sentinel-1A data offer advantages such as high resolution, global coverage, and short repeat observation periods. Its long-term operation provides rich data support for InSAR, making it suitable for analyzing long-term surface subsidence or tectonic activity. Therefore, Sentinel-1A image data and D-InSAR technology were utilized to obtain surface deformation information for the Huaxin Cement (Tibet) yard and the surrounding areas. The research utilized a total of eight scene images, which included four master images and four dependent images, spanning from 2019 to 2022.

Slope movement in the dump area during the annual rainy season was analyzed by pairing two images per year as differential interferometric pairs. The specific image data information is provided in Table 1.

**Table 1:** Sentinel-1A differential interference pair information.

Mater image	Auxiliary images	Time baseline/d	Position baseline/m	Incidence angle/^。^
2019/06/20	2019/08/31	72	−25.13	42.134
2020/06/26	2020/09/06	72	72.00	42.134
2021/07/03	2021/09/13	72	−41.51	42.134
2022/06/28	2022/**0**9/08	72	−82.34	42.134

### Deformation rate monitoring data selection

The data for this research consist of ascending orbit slant-range single-view complex image data acquired in the 60-scenario interferometric wide mode with VV polarization, spanning from November 4, 2018, to October 2, 2022. The image details are provided in Table 2. Additionally, satellite precise orbit ephemerides were used to enhance the processing accuracy of the satellite data. The SAR data from November 17, 2020, served as the master image in the analysis.

**Table 2:** Sentinel-1A 60 scene data information.

Number	Imaging time	Number	Imaging time	Number	Imaging time	Number	Imaging time
1	2018/11/04	2	2018/11/28	3	2018/12/22	4	2019/01/15
5	2019/02/08	6	2019/03/04	7	2019/03/28	8	2019/04/21
9	2019/05/15	10	2019/06/08	11	2019/07/02	12	2019/07/26
13	2019/08/19	14	2019/09/12	15	2019/10/06	16	2019/10/30
17	2019/11/23	18	2019/12/17	19	2020/01/10	20	2020/02/03
21	2020/02/27	22	2020/03/22	23	2020/04/15	24	2020/05/09
25	2020/06/02	26	2020/06/26	27	2020/07/20	28	2020/08/13
29	2020/09/06	30	2020/09/30	31	2020/10/24	32	2020/11/17
33	2020/12/11	34	2021/01/04	35	2021/01/28	36	2021/02/21
37	2021/03/17	38	2021/04/10	39	2021/05/04	40	2021/05/28
41	2021/06/21	42	2021/07/15	43	2021/08/08	44	2021/09/01
45	2021/09/25	46	2021/10/19	47	2021/11/12	48	2021/12/06
49	2021/12/30	50	2022/01/23	51	2022/02/16	52	2022/03/12
53	2022/04/05	54	2022/04/29	55	2022/05/23	56	2022/06/16
57	2022/07/22	58	2022/08/15	59	2022/09/08	60	2022/10/02

## Methods: InSAR monitoring mechanism for high-altitude dams

### Deformation monitoring of high-altitude dumps

Deformation monitoring and evaluation of high-altitude dumps during the rainy season were carried out to assess the extent of disaster risks caused by rainfall and to provide support for disaster prevention and mitigation in these high-altitude areas. Conventional methods currently in use have several limitations, such as insufficient resolution and an inability to perform large-scale monitoring. Synthetic Aperture Radar Differential Interferometry (D-InSAR) technology can overcome these challenges, making it an ideal tool for monitoring high-altitude dumps.

**Fig 5:**
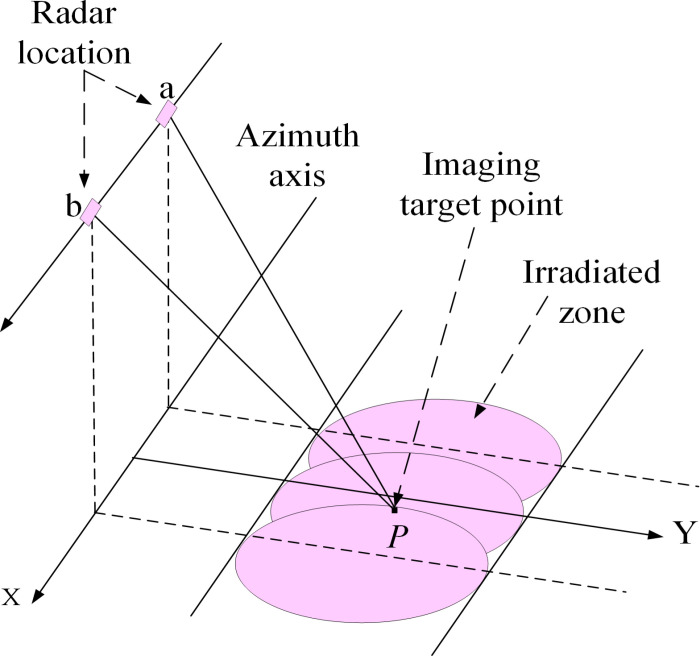
D-InSAR basic principle.

D-InSAR is based on radar interferometry techniques. When combined with existing high-precision Digital Elevation Model (DEM) data, it can eliminate the terrain phase effect in interferograms, enabling the monitoring of small deformations. The schematic diagram of D-InSAR, shown in Fig 5, illustrates that the dual-scene data used in D-InSAR processing consists of one scene before deformation and one scene after deformation. In the diagram, *a* and *b* denote the two positions of the radar, *P* represents a stationary observation point on the surface, and the pink circle indicates the radial range of the radar signal, which covers the observation area on the surface. Differential interferograms are generated from the two-scene data, and the surface deformation information is extracted from these interferograms.

D-InSAR uses repeated orbital interferometry and the composition of the interference phase in the interference fringes of the two-image data is given by:


$$\phi_{1}\text{=}-\frac{4\pi }{\lambda }R_{1}$$ (1)

$$\phi_{2}\text{=}-\frac{4\pi }{\lambda }R_{\text{2}}$$ (2)

where $\phi_{\text{1}}$, $\phi_{\text{2}}$ is the interference phase; *λ* is the radar wavelength (m); *R*_1_ is the distance from a to point *P* (m); *R*_2_ is the distance from b to point *P* (m).


$$△\phi \text{=}\phi_{2}-\phi_{1}=-\frac{4\pi }{\lambda }\left(R_{2}-R_{1}\right)=-\frac{4\pi }{\lambda }\delta r$$ (3)

where δr is the deformation.

### Deformation rate monitoring of high-altitude dumps

By monitoring the deformation rate of the high-altitude dumps during the rainy season, the deformation trend of the dump slopes can be inferred, helping to reduce disaster risks. Since SBAS-InSAR can monitor detailed surface deformation changes, this study employs SBAS-InSAR to monitor the slope deformation rate of high-altitude dumps.

SBAS-InSAR combines SAR data from multiple scenes within the same region to generate short baseline interferogram pairs. Using the principle of short baseline, singular value decomposition is applied to generate the average deformation rate map and the time series of coherent points. Differential interferograms are then produced to obtain the interference phase, enabling the analysis of surface deformation.

According to the time series, $t_{0}$, $t_{\text{1}}$, …, $t_{N}$, *N* + 1, single-view complex SAR data images are obtained, and one SAR data image is selected as the super main image. Then, image registration is performed and the vertical baseline thresholds are set. The SAR data images whose vertical baselines are less than the thresholds are merged into one group, with *L* groups in total.

Differential interferometric processing is performed on each group of SAR images to form *L* groups of images,

Each group of SAR images is processed by differential interference to form L groups of images and obtains *M* differential interferogram. If *N* is an odd number, *M* of the differential interferograms is:


$$\frac{N+1}{2}\leq M\leq N\left(\frac{N+1}{2}\right)$$ (4)

Taking $t_{0}$ as the initial time, the differential phase $\phi \left(t_{i},x,r\right)$ of the pixel $\left(x,r\right)$ in the differential interferogram relative to $t_{0}$ at any moment, $t_{i}$(*i* = 1, 2, …, *N*) is an unknown parameter, and the monitoring quantity is the differential interference phase $\delta \phi_{k}\left(x,r\right)$ (*k* = 1, 2,…, *M*) obtained by data processing.

For each pixel $\left(x,r\right)$ in the *k*^th^ (*k* = 1, 2, …, *M*) amplitude differential interference phase diagram, the following equation can be formed:


$$\delta \phi_{k}\left(x,r\right)=\phi \left(t_{B},x,r\right)-\phi \left(t_{A},x,r\right)=\frac{4\pi }{\lambda }\left[d\left(t_{B},x,r\right)-d\left(t_{A},x,r\right)\right]+\phi_{topo}\left(x,r\right)+\phi_{orb}+\phi_{res}\left(x,r\right)$$ (5)

where: *λ* is the radar wavelength; $d\left(t_{A},x,r\right)$ and $d\left(t_{B},x,r\right)$ are the surface deformation in the LOS direction of $t_{A}$ to $t_{B}$ pixel $\left(x,r\right)$, respectively, $\phi_{topo}(x,r)$ is the terrain phase caused by DEM data; $\phi_{orb}$ is the orbital phase caused by inaccurate SAR image data; and $\phi_{res}(x,r)$ is the residual phase.

Given the distinct and abrupt alternation between dry and wet seasons in high-altitude regions, the deformation monitoring model must account for various influencing factors. To address this, we combined meteorological factors, specifically rainfall and temperature, to improve the InSAR deformation monitoring model. This modification eliminates the impact of inter-seasonal variations on the calculation of SBAS-InSAR data, thereby enhancing the overall accuracy of the deformation analysis.

For temperature and rainfall, the following deformation models are used:


$$d_{1}=\beta_{1}T\left(t\right)+\beta_{2}P\left(t\right)$$ (6)

where: *d*_1_ is the surface deformation related to external environmental factors; *T*(*t*) and *P*(*t*) are the temperature and precipitation corresponding to time *t*; $\beta_{1}$ and $\beta_{2}$ are the coefficients of temperature and precipitation.

The deformation caused by internal factors adopts the 4th degree polynomial deformation model:


$$d_{2}=\sum_{1}^{4}\mu_{p}t^{p}$$ (7)

where: *d*_2_ is the surface deformation related to internal factors; *p* is the exponential of the accumulation time *t* (*p* = 1, 2, 3, 4); $\mu_{p}$ are the linear velocity, acceleration, and third-order velocity terms in the fourth-order polynomial deformation model, four speed items.

Therefore, considering temperature and rainfall, the following deformation model is constructed:


$$D=\sum_{1}^{4}\mu_{p}t^{p}+\beta_{1}T\left(t\right)+\beta_{2}P\left(t\right)$$ (8)

Considering that this research focuses on a slope within a high-altitude artificial accumulation field, no consideration is given to phase errors caused by DEM data; instead, precise orbit SAR images are selected for this project.

If the orbital phase is not considered, then the improved deformation model combined with equation (5) can be obtained:


$$\begin{array}{l} \delta \phi_{k}\left(x,r\right)=\phi \left(t_{B},x,r\right)-\phi \left(t_{A},x,r\right)\\ \approx \frac{4\pi }{\lambda }\left[\sum_{p=1}^{4}\mu_{p}\left(x,r\right)\left(t_{B}^{P}-t_{A}^{p}\right)+\beta_{1}\left(x,r\right)\left(T\left(t_{B}\right)-T\left(t_{A}\right)\right)+\beta_{2}\left(P\left(t_{B}\right)-P\left(t_{A}\right)\right)\right] \end{array}$$ (9)

$$C={\left[\mu_{1}\left(x,r\right),\mu_{2}\left(x,r\right),\mu_{3}\left(x,r\right),\mu_{4}\left(x,r\right),\beta_{1},\beta_{2}\right]}^{T}$$ (10)

BC=δϕ (11)

where: ***B*** is aN-1×6dimensional matrix, the form of matrix ***B*** in formula (11) is:


$$B=\left[\begin{array}{cccccc} t_{2}-t_{1} & t_{2}^{2}-t_{1}^{2} & t_{2}^{3}-t_{1}^{3} & t_{2}^{4}-t_{1}^{4} & T\left(t_{2}-t_{1}\right) & P\left(t_{2}-t_{1}\right)\\ t_{3}-t_{1} & t_{3}^{2}-t_{1}^{2} & t_{3}^{3}-t_{1}^{3} & t_{3}^{4}-t_{1}^{4} & T\left(t_{3}-t_{1}\right) & P\left(t_{3}-t_{1}\right)\\ \vdots & \vdots & \vdots & \vdots & \vdots & \vdots \end{array}\right]$$ (12)

If observation phaseδϕhas the same weight, the estimated value of ***C*** can be solved by the least square method:


$$C={\left(B^{T}B\right)}^{-1}B^{T}\delta \phi$$ (13)

Regarding phase unwrapping, atmospheric noise in high-altitude areas is relatively severe. To mitigate the effects of atmospheric noise caused by large topographic relief, multi-resolution analysis was applied. This approach reduces the influence of atmospheric noise by analyzing the interferometric phase before phase unwrapping through multi-resolution techniques.

Let *U*(*b*, *q*) be expressed as a two-dimensional m × n interference phase diagram after phase unwinding, and according to multi-resolution analysis, it is expressed as:


$$U\left(b,q\right)=\sum_{i_{x}}^{m-1}\sum_{j_{y}}^{n-1}v_{i_{x}j_{y}}\Phi_{Ji_{x}j_{y}}\left(b,q\right)+\sum_{j^{'}}^{J-1}\sum_{i_{x}}^{m-1}\sum_{j_{y}}^{n-1}w_{j^{'}}^{\varepsilon }\Psi_{j^{'}i_{x}j_{y}}^{\varepsilon }\left(b,q\right)$$ (14)

where: $\Phi_{j^{'}i_{x}j_{y}}\left(b,q\right)=\Phi_{Ji_{x}}\left(b\right)\cdot \Phi_{Ji_{y}}\left(q\right)$, $v_{j^{'}i_{x}j_{y}}=\left[\Omega \left(b,q\right),\Psi_{j^{'}i_{x}j_{y}}\left(b,q\right)\right]$, $w_{j^{'}i_{x}j_{y}}^{\varepsilon }=\left[\Omega \left(b,q\right),\Psi_{j^{'}i_{x}j_{y}}^{\varepsilon }\left(b,q\right)\right]$, $\Psi_{j^{'}i_{x}j_{y}}^{\varepsilon }\left(b,q\right)= \{\begin{array}{l} \Psi_{j^{'}i_{x}}^{\varepsilon }\left(b\right)\cdot \Phi_{ji_{y}}^{\varepsilon }\left(q\right),\varepsilon =1\\ \Phi_{j^{'}i_{x}}^{\varepsilon }\left(b\right)\cdot \Psi_{ji_{y}}^{\varepsilon }\left(q\right),\varepsilon =2\\ \Psi_{j^{'}i_{x}}^{\varepsilon }\left(b\right)\cdot \Phi_{ji_{y}}^{\varepsilon }\left(q\right),\varepsilon =3 \end{array}$.

where: *Φ* and *Ψ* are translation function and generating wavelet function respectively; *v* and *w* are the low frequency and high frequency coefficients of wavelet; *J* is the wavelet decomposition scale; The value of *ε* are 1, 2, and 3, which indicate three directions, namely horizontal, vertical, and diagonal.

**Fig 6:**
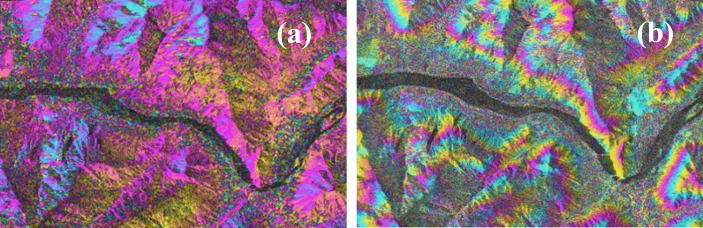
Improved before-and-after charts: (a) Before improved, (b) After improvement.

Using data from two scenes in the study area, a set of interferograms was generated by comparing the improved deformation monitoring model with the unimproved model, as shown in Fig 6.

As illustrated in Fig 6, the improved deformation monitoring model effectively reduces the impact of temperature-induced atmospheric noise in high-altitude regions. The results show that, compared to the unimproved interferograms, the improved interferograms exhibit clearer interference fringes, indicating a more accurate representation of surface deformation.

### The relationship between the deformation of dump slope and the deformation of InSAR LOS

**Fig 7:**
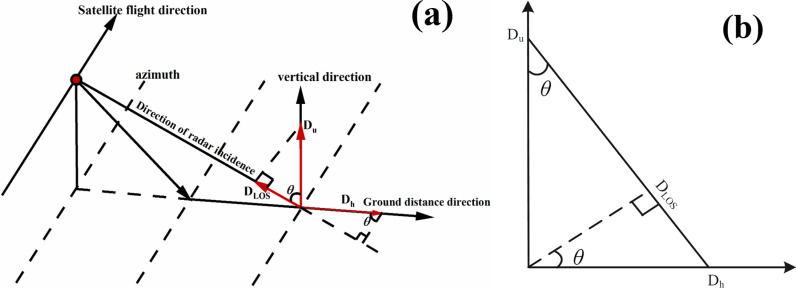
Relationship between SAR satellite LOS direction and surface deformation.


Based on the InSAR monitoring principle for high-altitude dump slopes, the Line-of-Sight (LOS) deformation information is obtained. Considering the geometric relationship between SAR imaging and the ground (Fig 7), the LOS deformation from the SAR satellite can be transformed into vertical deformation and ground distance deformation. Fig 7b illustrates the triangular relationship among the LOS direction deformation, vertical direction deformation, and ground distance direction deformation, with all three being mutually perpendicular to one another. The relationship between LOS direction deformation information and ground distance direction$D_{h}$as well as vertical direction$D_{u}$can be expressed as follows:

$$D_{LOS}=D_{u}cos\theta +D_{h}sin\theta$$ (15)

where:$D_{LOS}$is the orbital elevation LOS deformation vector;$D_{u}$is the vertical component of $D_{LOS}$;$D_{h}$is the component of$D_{LOS}$in the ground direction; *θ* is the radar incidence angle of InSAR in degrees.

The vector relationship among LOS direction deformations, vertical surface deformations, and ground distance surface deformations of SAR satellite can be described accordingly:


$$D_{LOS}=\left[\begin{array}{cc} cos\theta & sin\theta \end{array}\right]\left[\begin{array}{c} D_{u}\\ D_{h} \end{array}\right]$$ (16)

For this study's purpose, only considering vertical deformation information of dump slope while neglecting surface deformations in ground distance directions allows obtaining such information through following formula.


$$D_{u}=\frac{D_{LOS}}{cos\theta }$$ (17)

### Verification of InSAR monitoring accuracy at high-altitude

In this study, Trimble’s RTK technology was employed to obtain highly accurate positioning results efficiently. The device supports both GPS and GLONASS satellite systems and provides high-precision real-time kinematic (RTK) positioning, with a horizontal accuracy of ±10mm in RTK mode and a vertical accuracy of ±20mm. Differential data were transmitted in real-time between the reference and mobile stations, followed by quality checks and preliminary processing using Trimble Access software.

The accuracy of InSAR technology was statistically analyzed by comparing the deformation results obtained from GNSS RTK technology monitoring at the discharge site.

GNSS RTK technology was used to monitor the discharge site at the southern end of the Shanan Huaxin Cement mine. This setup includes one high-precision real-time automatic monitoring benchmark station and six high-precision real-time automatic monitoring observation stations, totaling seven stations, for the monitoring period from January 10, 2022, to July 23, 2022. The exact locations of the six monitoring points are shown in Table 4.

**Fig 8:**
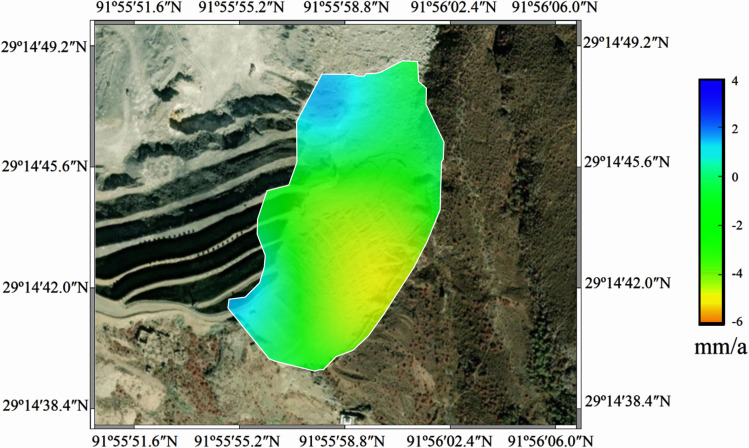
Deformation rate diagram.

A total of 17 Sentinel-1A satellite images from January 11, 2022, to August 3, 2022, were utilized for SBAS-InSAR calculations. Specific details of the image data are provided in Table 3, and Fig 8 illustrates the deformation results map.

**Table 3:** Sentinel-1A image data information.

No.	Image date	No.	Image date	No.	Image date	No.	Image date	No.	Image date
1	2022/01/11	2	2022/01/23	3	2022/02/04	4	2022/02/16	5	2022/02/28
6	2022/03/12	7	2022/03/24	8	2022/04/05	9	2022/04/17	10	2022/04/29
11	2022/05/11	12	2022/05/23	13	2022/06/04	14	2022/06/16	15	2022/06/28
16	2022/07/22	17	2022/08/03						

**Table 4:** Monitoring point deformation rate values.

Point	Deformation rate/mm·a^−1^	Point	Deformation rate/mm·a^−1^	Point	Deformation rate/mm·a^−1^
91°55′58.8″E 29°14′40.9″N	**−**3.9	91°55′59.9″E 29°14′43.1″N	**−**4.4	91°56′01.3″E 29°14′45.6″N	**−**1.0
91°55′55.9″E 29°14′42.4″N	0.2	91°55′57.0″E 29°14′44.5″N	**−**2.3	91°55′58.4″E 29°14′46.7″N	1.0

**Fig 9:**
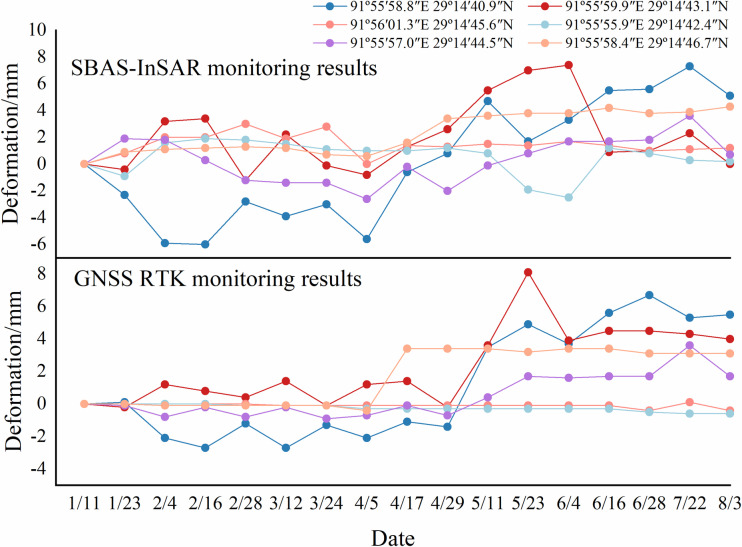
Comparison of GNSS RTK and SBAS-InSAR data.

Table 4 presents the deformation rates for each monitoring point. Time series data extracted from both GNSS RTK and SBAS systems for the six monitoring points are shown in Fig 9 for comparison.

**Fig 10:**
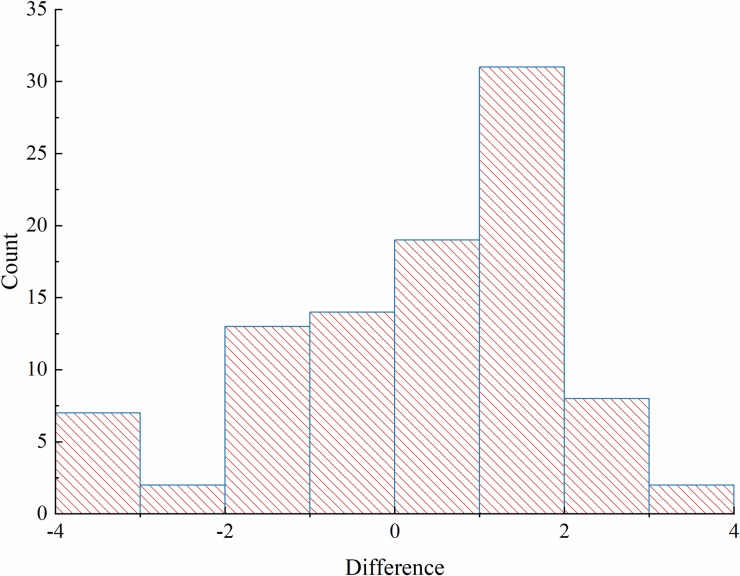
Difference distribution plot.

The InSAR and GNSS RTK time series data are analyzed using the analysis above (Table 5), and the distribution of differences is shown in Fig 10.

**Table 5:** InSAR and GNSS RTK time series differences (Unit: mm).

Point	91°55′58.8″E 29°14′40.9″N	91°55′59.9″E 29°14′43.1″N	91°56′01.3″E 29°14′45.6″N	91°55′55.9″E 29°14′42.4″N	91°55′57.0″E 29°14′44.5″N	91°55′58.4″E 29°14′46.7″N
Date						
2022/01/23	**−**2.4	**−**0.2	0.8	**−**0.9	2.0	0.9
2022/02/04	**−**3.8	2.0	2.0	1.6	2.6	1.2
2022/02/16	**−**3.3	2.6	2.0	1.9	0.5	1.3
2022/02/28	**−**1.6	**−**1.6	3.0	1.9	**−**0.4	1.4
2022/03/12	**−**1.2	0.8	2.0	1.6	**−**1.2	1.3
2022/03/24	**−**1.7	0.0	2.9	1.2	**−**0.5	0.8
2022/04/05	**−**3.5	**−**2.0	0.1	1.3	**−**1.9	1.0
2022/04/17	0.5	**−**0.1	1.5	1.3	**−**0.1	**−**1.8
2022/04/29	2.2	2.8	1.4	1.5	**−**1.3	0.0
2022/05/11	1.2	1.9	1.6	1.1	**−**0.5	0.2
2022/05/23	**−**3.2	**−**1.1	1.5	**−**1.6	**−**0.9	0.6
2022/06/04	**−**0.4	3.5	1.8	**−**2.2	0.1	0.4
2022/06/16	**−**0.1	**−**3.6	1.5	1.5	0.0	0.8
2022/06/28	**−**1.1	**−**3.5	1.4	1.3	0.1	0.7
2022/07/22	2.0	**−**2.0	1.0	0.9	0.0	0.8
2022/08/03	**−**0.4	**−**4.0	1.6	0.8	**−**1.0	1.2

In this study, a paired sample t-test analysis was conducted to examine the relative deviation between GNSS RTK and SBAS-InSAR techniques. Since both GNSS RTK and SBAS-InSAR data were obtained from the same measurement points, they form paired observations that meet the requirements of this method. Compared to other approaches (e.g., independent sample t-test or non-parametric tests), the paired t-test capitalizes on the relationship between paired data, enhancing statistical efficiency and making it more suitable for assessing significant differences between the two techniques. The process of the paired sample t-test includes calculating the difference for each pair of data, followed by a normality test:

(1) Establish test hypotheses, determine the test level, take α=0.05.

$H_{\text{0}}:\mu_{d}\text{=0}$, the difference between GNSS RTK and SBAS-InSAR monitoring slope deformation information of high-altitude dump is 0;
$H_{\text{0}}:\mu_{d}\neq \text{0}$, the difference between GNSS RTK and SBAS-InSAR monitoring slope deformation information of high-altitude dump is not 0;

(2) Calculate test statistics:

$$\bar{d}=\frac{\sum d}{n}\text{=}\frac{\sum_{i=1}^{96}d_{i}}{\text{96}}=0.2\text{8}$$ (18)

$$S_{\bar{d}}=\frac{S_{d}}{\sqrt{n}}=\frac{\sqrt{\frac{\sum_{i=1}^{n}{\left(d_{i}-\bar{d}\right)}^{2}}{n-1}}}{\sqrt{n}}=\frac{\sqrt{\frac{\sum_{i=1}^{96}{\left(d_{i}-0.2\text{8}\right)}^{2}}{96-1}}}{\sqrt{96}}=\frac{1.694}{9.798}=0.17$$ (19)

$$t=\frac{\bar{d}}{S_{\bar{d}}}=\frac{0.2\text{8}}{0.17}=1.6\text{5}$$ (20)

v=n−1=96−1=95 (21)

where: $H_{0}$ is the original hypothesis; $H_{1}$ is the alternative hypothesis; *α* is the confidence level; *d* is difference for each group; $\bar{d}$ is the overall mean of the difference in each group; *n* is a statistic; $S_{\bar{d}}$ is the standard error of the mean value of the difference; *v* is freedom.

By looking up the (*t*-limit table) and obtaining $t_{0.05/2,95}=\text{1.99}$, i.e., $t=1.6\text{5}<t_{0.05/2,95}=1.9\text{9}$, it indicates that there is no statistically significant difference between GNSS RTK and SBAS-InSAR in terms of their ability to monitor deformation information accurately, meeting engineering requirements.

The differences between InSAR monitoring results and GNSS monitoring results are evaluated by statistical mean error *θ* analysis. The mean error and standard deviation are calculated as:


$$\theta \text{=}\frac{\text{1}}{N}\sum_{i=1}^{N}\left(\left|d_{\text{InSAR}}^{i}\right|\text{-}\left|d_{\text{GNSS}}^{i}\right|\right)$$ (22)

where: *d*_InSAR_ is the InSAR monitoring result; *d*_GNSS_ is the GNSS RTK monitoring result; *N* is the number of points.

The mean error *θ* in this study area is 0.28 mm

## Results

### Deformation index evaluation

**Fig 11:**
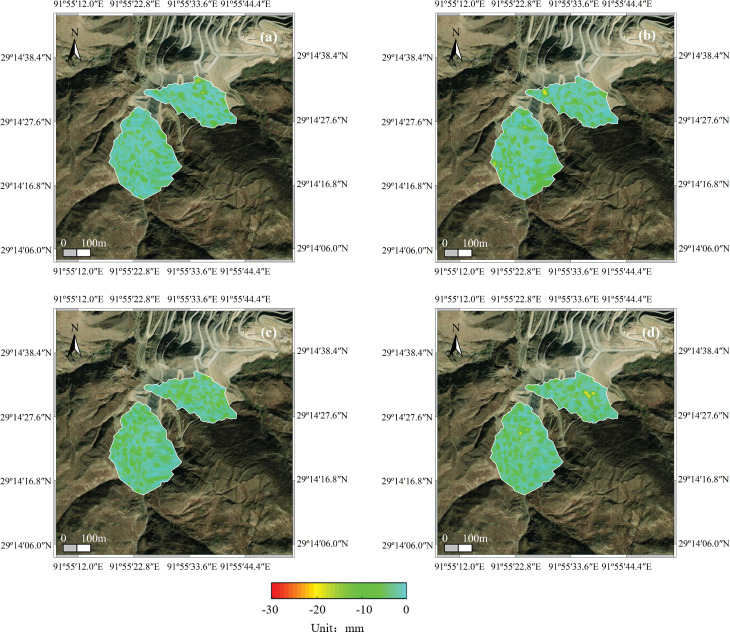
Deformation data vector map per year: (a) in 2019, (b) in 2020, (c) in 2021, (d) in 2022.

The deformation map of the study area for each year from 2019 to 2022 is shown in Fig 11. Negative deformation values indicate subsidence, while positive values indicate uplift.

As seen in Fig 11, the deformation of the #2 Dump during the rainfall concentration periods in 2020, 2021, and 2022 is larger, whereas the deformation during the rainfall period in 2019 is relatively minor. To quantitatively analyse the deformation characteristics for each year, the maximum deformation value for the entire dump area was extracted, as shown in Table 6.

**Table 6:** Maximum deformation values for the rainy season from 2019 to 2022.

Year	Dump	Longitude/°	Latitude/°	Maximum deformation values/mm
2019	#1	91°55′24.60″	29°14′17.88″	**−**9.6
	#2	91°55′35.04″	29°14′34.44″	**−**15.9
2020	#1	91°55′18.12″	29°14′20.76″	**−**17.2
	#2	91°55′28.20″	29°14′33.00″	**−**20.1
2021	#1	91°55′22.44″	29°14′21.12″	**−**8.3
	#2	91°55′32.20″	29°14′34.44″	**−**16.5
2022	#1	91°55′23.52″	29°14′25.08″	**−**17.2
	#2	91°55′36.48″	29°14′31.92″	**−**19.4

**Fig 12:**
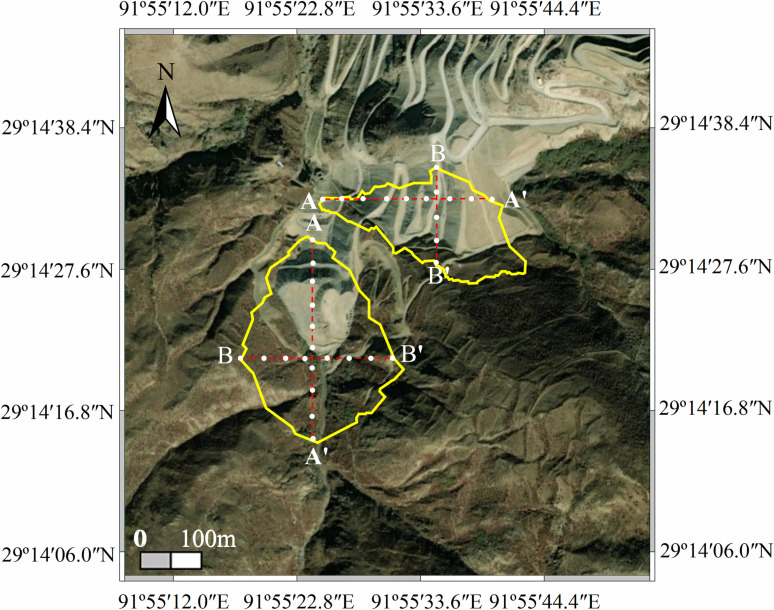
Distribution map of isometric control points in the dump.

**Fig 13:**
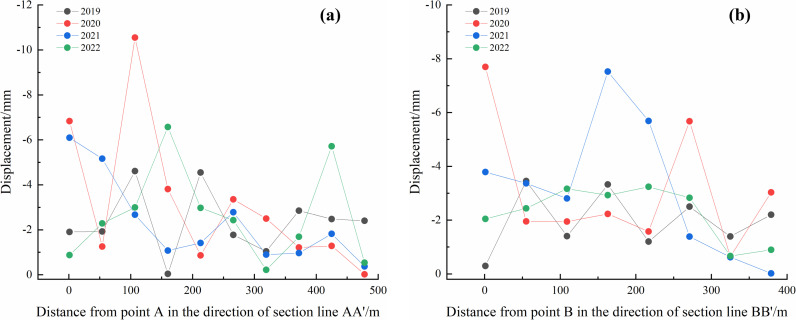
Deformation variation curves of control points in #1 Dump: (a) AA’ direction each year and (b) BB’ direction every year.

**Fig 14:**
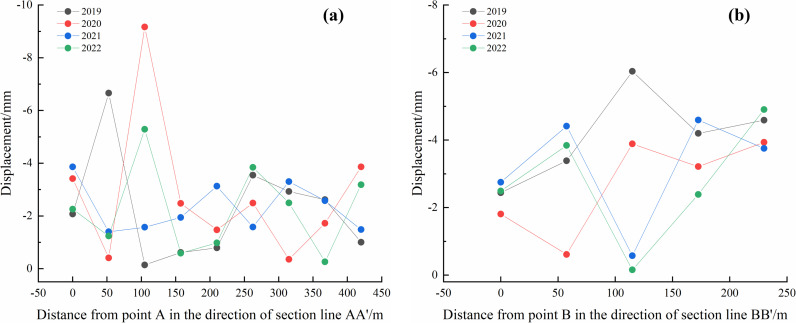
Deformation analysis curves of control points in #2 Dump: (a) AA’ direction each year and (b) BB’ direction every year.

The deformation data from the annual rainfall concentration periods were extracted, and equidistant reference points for each year were arranged along section lines AA’ and BB’ (Fig 12). The settlement deformation information at these control points was analyzed, as shown in Figs 13 and 14.

Fig 13 illustrates that for the #1 Dump, deformation in the profile direction during the rainfall concentration periods in 2019 and 2022 ranges from 0 to **−**5mm. In 2020, deformation fluctuated significantly in the profile direction, with the maximum deformation exceeding **−**10mm. In 2021, the deformation ranged from 0 to **−**8 mm during the rainfall concentration period.

Fig 14 shows that for the #2 Dump, the deformation in the profile direction during the 2019 rainy season ranges from 0 to **−**8 mm. In the 2020 rainy season, the maximum deformation in the profile direction is 0 to **−**10 mm. In the 2021 and 2022 rainy seasons, the maximum deformation in the profile direction is 0 to **−**5 mm.

Based on the above analysis, the maximum deformation exceeds 20 mm, with the deformation degree from 2020 reaching the Blue Alert Threshold according to the single landslide warning criterion [19] (Table 7).

**Table 7:** Single warning criteria for landslides in dumps.

Monitoring Indicators/ Alert Threshold	Red	Orange	Yellow	Blue	Max	
					#1 Dump	#2 Dump
**Vertical Displacement/mm**	80	40	30	20	**−**17.2	**−**20.1

### Deformation rate index evaluation

**Fig 15:**
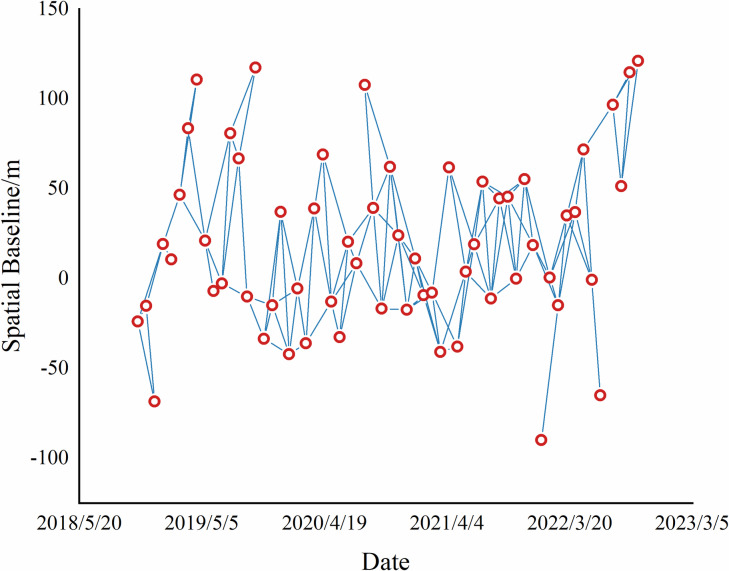
Spatial-temporal distribution.

The 60-scene data were processed by selecting one scene as the master image and using the remaining 59 scenes as auxiliary images. The optimal pairing was conducted, resulting in a maximum of 1,770 pairs of interferograms. The spatio-temporal distribution of the interferograms is shown in Fig 15.

The study area is in a high-altitude region, where significant and abrupt transitions between dry and wet seasons occur. To account for the influence of temperature and rainfall across these seasonal transitions, the SBAS-InSAR calculation was adjusted to eliminate seasonal effects, thereby improving the overall accuracy of the deformation measurements. By incorporating temperature and rainfall data for the study area, a differential interferogram was generated.

**Fig 16:**
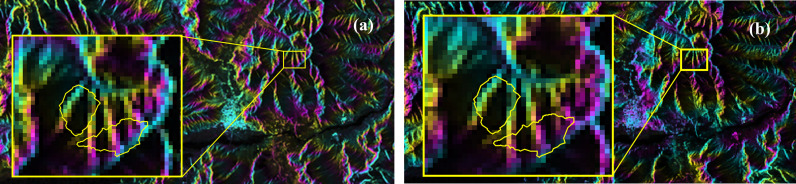
Interferogram after replanning: (a) 2019/03/04–2019/04/21 (b) 2019/03/04–2019/05/15.

Coherent target extraction, de-levelling, discrete point unwrapping, re-delevelling, and registration with the super master image were performed on all the generated data pairs. The re-flattened results were then geocoded to correspond with the study area, as shown in Fig 16.

**Fig 17:**
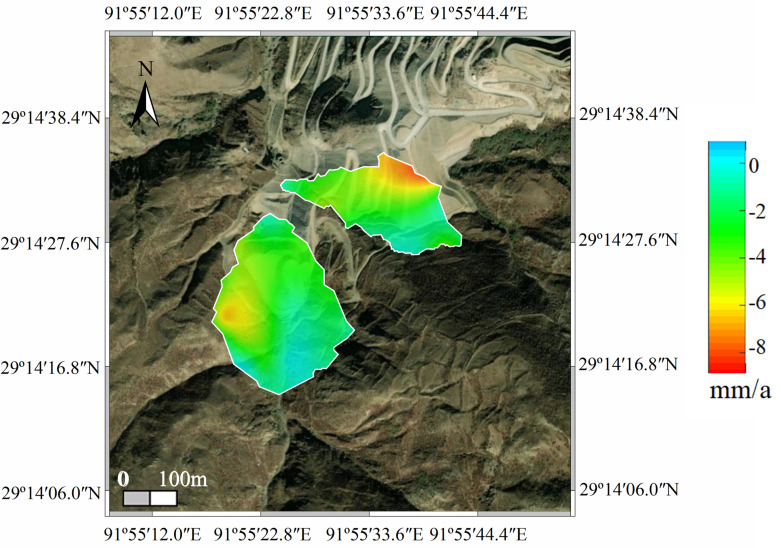
Deformation rate map of the study area.

The spatial distribution of surface deformation was obtained using SBAS-InSAR technology, producing a deformation rate map for the period from November 4, 2018, to October 2, 2022 (Fig 17). The deformation distribution in the study area showed significant variation, with a predominantly negative deformation rate, indicating subsidence. From 2018 to 2022, only a small number of deformation areas were identified, and the annual average deformation rate ranged from 0 to **−**9.00 mm/a.

**Fig 18:**
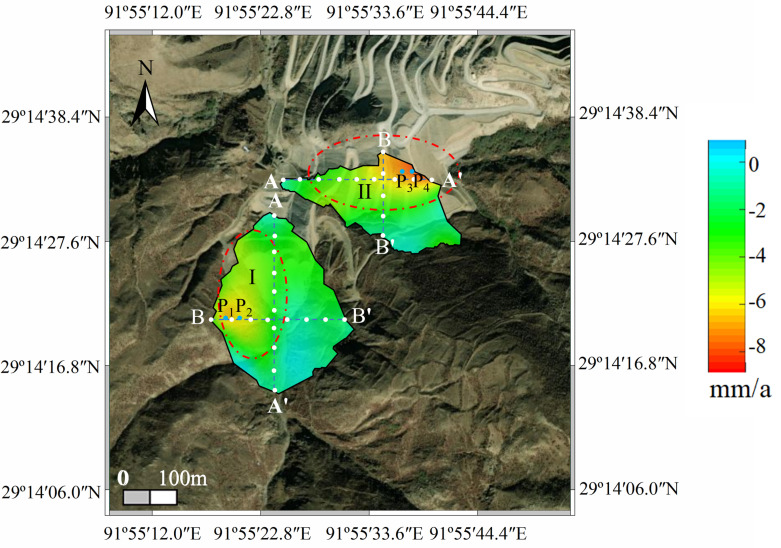
Deformation rate analysis diagram of the dump.

**Fig 19:**
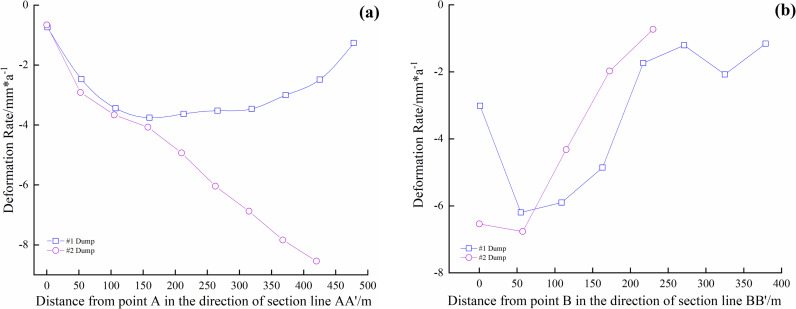
Deformation rate curves in the direction of section line of soil dump. (a) AA’ of #1 and #2 Dumps. (b) BB’ of #1 and #2 Dumps.

For the average deformation rate map shown in Fig 18, equidistant control points were arranged along the section lines AA’ and BB’ of the dump. Extraction points P1–P4 were placed within the deformation areas, and the regions of maximum deformation (I and II) within the study area were also identified. Fig 18 illustrates the deformation rates of the equidistant control points along the section lines, while Fig 19 shows the displacement of points P1–P4 over time.

Fig 19 shows that the main deformation area of #1 Dump is located in the upper-left section, with the deformation rate along the AA’ direction varying significantly. The deformation rate ranges from 0 to **−**4.0 mm/a and exhibits a “V”-shaped distribution along AA’, while in the BB’ direction, the deformation shows an oscillating distribution, ranging from 0 to **−**6.0 mm/a. The main deformation area of #2 Dump is in the lower section. In the AA’ direction, the deformation rate near Point A shows a downward trend, with a higher deformation rate near A’ (around **−**9.0 mm/a). In the BB’ direction, the deformation rate increases.

Based on the above analysis, the maximum deformation rate of the dump does not exceed 1.0 m/a. Using the landslide classification based on sliding velocity [20] (Table 8), the dump slope falls under category VII, indicating a very slow potential landslide state. No slippage has occurred on the dump's slope to date.

**Table 8:** Classification of landslides based on sliding velocity.

Category	Landslide Sliding State	Typical Speed	Maximum Deformation Rate of Dumps	
			#1 Dump	#2 Dump
Ⅰ	super-fast	>5 m/s	–	–
Ⅱ	soon	>5 m/min	–	–
Ⅲ	fast	>1 m/h	–	–
Ⅳ	medium speed	>10 mm/month	–	–
Ⅴ	slow	>1 m/a	–	–
Ⅵ	very slow	>10 mm/a	–	–
Ⅶ	super slow	<10 mm/a	**−**6.28mm/a	**−**8.03mm/a

## Discussion

**Fig 20:**
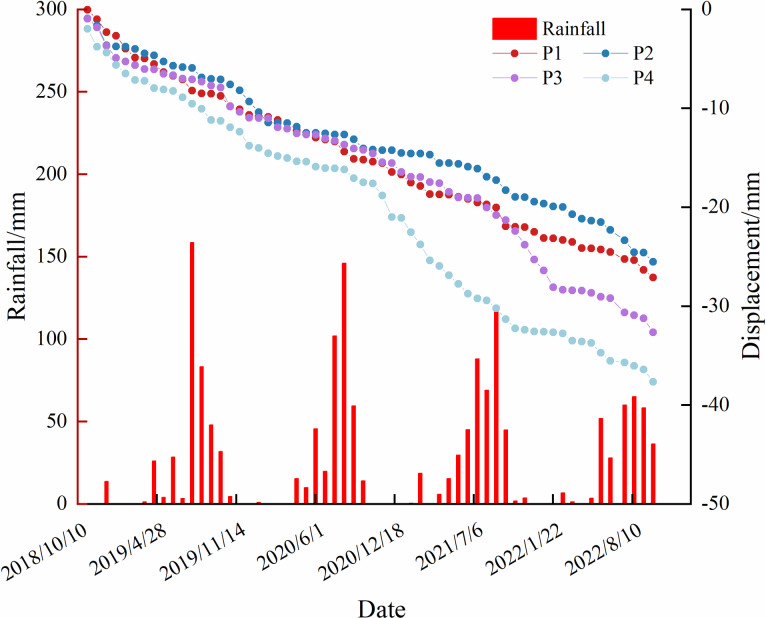
P_1_-P_4_ time deformation series with corresponding temporal rainfall plot.

Combining the temporal deformation results from monitoring points P1–P4 with the rainfall data for the corresponding periods, Fig 20 was produced.

Fig 20 illustrates that the rainfall in the study area is typically concentrated between May and August, with reduced rainfall from September to December and January to May. The time series analysis shows a partial correlation between deformation and rainfall; however, the deformation displacement curve does not always follow rainfall patterns. From Fig 20, we observe stable deformation periods during dry seasons, such as from November 2018 to April 2019, September 2019 to April 2020, and September to November 2021. A peak in rainfall from May to August 2019 led to a sudden increase in deformation, whereas deformation remained stable despite the concentrated rainfall from May to August 2020. However, during the rainy season from August to September 2021, deformation increased sharply.

While the dump's slope is impacted by rainfall, there is a delay between the rainfall and the onset of deformation. For instance, although rainfall peaked from July to August 2019, the corresponding deformation did not show significant change immediately; however, there was considerable subsidence in August to September. Similarly, the dry period from September 2020 to April 2021 saw an increase in slope deformation, likely due to rainfall during the previous rainy season.

Overall, the slope experienced significant settling during the rainy season, while it remained relatively stable during the dry season. However, slope deformation and displacement in the dumps do not fully correlate with rainfall patterns, indicating a lag effect in the deformation process. The deformation sequence in Fig 20 shows that slope deformation accelerates approximately two months after concentrated rainfall.

Many studies have highlighted the strong correlation between slope deformation and precipitation [21]. Changes in rainfall are known to be a key factor influencing slope deformation. For example, Zhou et al. observed a lag effect in subsidence changes during their research on the stability of slopes affecting high-speed railways [22]. Similarly, Cenni et al. found that sliding movements tend to accelerate 3–4 months after heavy rainfall [18]. Our findings align with these studies, showing a notable lag effect in subsidence changes.

## Conclusions

Taking the limestone dumps in Sangri County, Shannan, Tibet as the research object, based on InSAR technology, considering the impact of rainfall and temperature on the slope of limestone dump in high altitude area, and the monitoring accuracy of dump slope in high altitude area is tested by combining GNSS RTK monitoring. Furthermore, according to the deformation rate of the high-altitude dump slope, the stability of the dump slope is analyzed and the safety evaluation is carried out.

1) The InSAR monitoring deformation model was enhanced by incorporating meteorological factors such as rainfall and temperature. After correcting for InSAR monitoring accuracy using GNSS RTK data, the average error between GNSS RTK and SBAS-InSAR was found to be 0.28 mm, confirming the applicability of InSAR for monitoring high-altitude dump slopes.
2) Monitoring of the high-altitude dump slope during the rainy season revealed that heavy rainfall leads to overall settlement. If rainfall exceeds 300 mm and slope deformation surpasses 20 mm, it triggers the single warning threshold for landslide risk. The slope showed significant settling during the rainy season but remained stable in the dry season. However, there was a delay in deformation relative to rainfall, with a hysteresis phenomenon observed, where deformation peaked after the rainfall period.
3) The deformation rate of the high-altitude dump from 2018 to 2022 ranged from 0 to **−**9.00 mm/a, suggesting a relatively stable sliding trend, categorized as slip category VII. The potential landslide movement is very slow, with no significant slippage observed on the dump slope.

While this study successfully monitored and evaluated the stability of the high-altitude dump slope using SBAS-InSAR, and validated the monitoring results with GNSS data, some limitations remain. The accuracy of the SBAS-InSAR method can be influenced by factors such as baseline length and viewing geometry, leading to potential localized errors. The spatial distribution and temporal intervals of GNSS stations may also limit their representativeness across the entire slope, especially in remote or difficult-to-access areas where monitoring points may not fully capture subsidence variations.

Given the complex topography and climatic conditions of the high-altitude region, future research should focus on integrating multiple monitoring methods, such as combining ground-based observations, UAV monitoring, and geological surveys, to enhance the comprehensive understanding of slope deformation and improve early warning systems.
